# Overexpression of FTO inhibits excessive proliferation and promotes the apoptosis of human glomerular mesangial cells by alleviating FOXO6 m6A modification via YTHDF3-dependent mechanisms

**DOI:** 10.3389/fphar.2023.1260300

**Published:** 2023-09-26

**Authors:** Xingxing Zhuang, Tao Liu, Liangbing Wei, Jiarong Gao

**Affiliations:** ^1^ Department of Pharmacy, Chaohu Hospital of Anhui Medical University, Chaohu, China; ^2^ Department of Pharmacy, The First Affiliated Hospital of Anhui University of Chinese Medicine, Hefei, China

**Keywords:** FTO, FOXO6, m6A modification, glomerular mesangial cells, cell proliferation, cell apoptosis

## Abstract

**Background:** N6-methyladenosine (m6A) is a prevalent post-transcriptional modification presented in messenger RNA (mRNA) of eukaryotic organisms. Chronic glomerulonephritis (CGN) is characterised by excessive proliferation and insufficient apoptosis of human glomerular mesangial cells (HGMCs) but its underlying pathogenesis remains undefined. Moreover, the role of m6A in CGN is poorly understood.

**Methods:** The total level of m6A modification was detected using the m6A quantification assay (Colorimetric). Cell proliferation was assessed by EdU cell proliferation assay, and cell apoptosis was detected by flow cytometry. RNA sequencing was performed to screen the downstream target of fat mass and obesity-associated protein (FTO). MeRIP-qPCR was conducted to detect the m6A level of forkhead box o6 (FOXO6) in HGMCs. RIP assay was utilized to indicate the targeting relationship between YTH domain family 3 (YTHDF3) and FOXO6. Actinomycin D assay was used to investigate the stability of FOXO6 in HGMCs.

**Results:** The study found that the expression of FTO was significantly reduced in lipopolysaccharide (LPS)-induced HGMCs and renal biopsy samples of patients with CGN. Moreover, FTO overexpression and knockdown could regulate the proliferation and apoptosis of HGMCs. Furthermore, RNA sequencing and cellular experiments revealed FOXO6 as a downstream target of FTO in regulating the proliferation and apoptosis of HGMCs. Mechanistically, FTO overexpression decreases the level of FOXO6 m6A modification and reduces the stability of FOXO6 mRNA in a YTHDF3-dependent manner. Additionally, the decreased expression of FOXO6 inhibits the PI3K/AKT signaling pathway, thereby inhibiting the proliferation and promoting apoptosis of HGMCs.

**Conclusion:** This study offers insights into the mechanism through which FTO regulates the proliferation and apoptosis of HGMCs by mediating m6A modification of FOXO6 mRNA. These findings also suggest FTO as a potential diagnostic marker and therapeutic target for CGN.

## 1 Introduction

Chronic glomerulonephritis (CGN) is a common clinical refractory kidney disease, characterised by proteinuria, haematuria and hypertension. The lack of typical clinical symptoms in the early stage poses a challenge to the early diagnosis and intervention of CGN in most patients (Sethi and Fervenza, 2019; AlYousef et al., 2020). In China, the prevalence of CGN is increasing annually and has become a major contributor to end-stage renal disease (ESRD). The current available treatments for CGN mainly involve lifestyle changes, immunosuppressive therapy, monitoring blood pressure, reducing proteinuria, and improving kidney function. However, these treatments have very limited clinical efficacy and potential drug adverse effects, which are unable to effectively prevent CGN from progressing to ESRD (Hu et al., 2020; Yang et al., 2020). Therefore, identifying new therapeutic targets and valuable diagnostic markers for CGN are urgently needed.

Glomerular mesangial cells (GMCs) are the main functional cells of the glomerulus, which play a critical role in ensuring mesangial matrix homeostasis, maintaining glomerular structural integrity and regulating glomerular filtration (Avraham et al., 2021). However, when stimulated by certain pathogenic factors, GMCs are activated, leading to excessive proliferation and inadequate apoptosis. Consequently, the excessive proliferation of GMCs results in glomerular expansion, mesangial matrix deposition, impaired glomerular function and ultimately, renal impairment (Zhao, 2019). The existing literature has demonstrated that the excessive proliferation and insufficient apoptosis of GMCs are the main pathological characteristics of CGN (Scindia et al., 2010).

N6-methyladenosine (m6A) modification, characterised by the methylation modification at the sixth N position of adenosine, is a prevalent epigenetic modification occurring in eukaryotic messenger RNA (mRNA). Sequencing analysis reveals that m6A modification is predominantly concentrated within a conserved RRACH motif (R = G/A, H = A/C/U) and preferentially occurs near the 3′-UTR region of mRNA (Sendinc and Shi, 2023). Furthermore, recent studies have demonstrated that m6A modification is a dynamic and reversible RNA modification process, which is primarily regulated by three main types of enzymes, namely, methylated transferase (including WTAP, METTL3 and METTL14), demethylated transferase (including ALKBH5 and FTO) and methylated recognition protein (including YTHDF1/2/3 and IGF2BP1/2/3) (Oerum et al., 2021). The m6A modification plays significant roles in various post-transcriptional regulation processes of RNA, including transcription, translation, stability, splicing, and localization. Studies have confirmed that m6A modification plays an crucial role in the occurrence and development of various kidney diseases, including diabetic nephropathy, acute kidney injury, and kidney cancer (Shen et al., 2021; Jiang et al., 2022; Wang et al., 2022). However, the potential role of m6A modification in the pathogenesis of CGN still remains understudied and unclear.

In this study, we attempted to explore the biological role of FTO-mediated m6A modification in regulating the proliferation and apoptosis of GMCs in CGN and revealed for the first time that FTO could potentially serve as a diagnostic marker and therapeutic target for CGN.

## 2 Materials and methods

### 2.1 Cell culture, cell lines, and grouping

The human glomerular mesangial cells (HGMCs, YS1702C) were acquired from Yaji Biological in Shanghai, China, and were cultured in Dulbecco’s Modified Eagle Medium (DMEM) with 10% fetal bovine serum (FBS) and 1% penicillin/streptomycin from Solarbio in Beijing, China, at 37°C in a 5% CO_2_ incubator.

According to the literature, lipopolysaccharide (LPS)-induced HGMCs were chosen as a model for cell proliferation in CGN studies (Liu et al., 2023; Zhuang et al., 2023), and the LPS used in this study was obtained from Solarbio Science & Technology Co., Ltd. (124S032, Beijing, China). The untreated HGMCs and LPS-induced HGMCs were considered as the control and model groups, respectively.

### 2.2 CCK8 cell viability assay

Cell viability was detected using CCK8 cell proliferation assay kit (BB19071X, Bestbio, Shanghai, China) following manufactory’s manual. The HGMCs were plated in 96-well plates and incubated at 37°C under 5% CO_2_. After cultivating for an appropriate time, 10 µL of CCK-8 was add to each well, mix gently, and incubate for an additional 1 h. Finally, the absorbance value of each well was measured at 450 nm using a microplate reader (RT6100, Rayto, United States) to calculate cell viability. All CCK-8 tests in each group were repeated three times.

### 2.3 Construction of the small interfering RNA (siRNA) and overexpression plasmid (OE)

The small interfering RNA (siRNA) targeting FTO and YTHDF3 for knockdown and the pcDNA3.1 plasmid vector for FTO and FOXO6 overexpression were synthesised by Anhui General Biological (Chuzhou, China). Both were transfected using Lipo 2000™ (11668019, Invitrogen, United States) into the cell lines following the manufacturer’s protocol.

### 2.4 m6A quantification assay (Colorimetric)

The EpiQuikTM m6A RNA Methylation Quantification Kit (Colorimetric) (2110044, Epigentek, United States) was used to calculate the amounts of RNA methylation (m6A). According to the manufacturer’s instructions, 200 ng of RNA, the capture antibody, and the detection antibody were added to each well. Based on the calorimetric examination of multiple incubations of m6A at 450 nm, a standard curve was created, and all tests were repeated three times.

### 2.5 Real-time quantitative polymerase chain reaction (RT-qPCR)

HGMCs’ total RNA was extracted using the TRIZOL reagent (248207, Life Technologies, United States), and cDNA was produced using PrimeScript RT Kit with gDNA Eraser (AJ51485A, Takara, Japan). RT-qPCR was carried out using the SYBR GREEN qPCR Master Mix (LT202201, Servicebio, Wuhan, China) on the ABI STEPOne Plus Real-Time PCR System (Applied Biosystems, United States). With β-actin serving as the internal reference, the primer sequences for PCR analysis are presented in the Supplementary Table S1. The 2^−ΔΔCT^ method was used to calculate the relative gene expression. The RT-qPCR experiments were replicated independently in six wells per sample.

### 2.6 Western blot

Using RIPA lysis buffer (69,128,033, Biosharp, Hefei, China), which contains 1 mM phenylmethylsulfonyl fluoride (PMSF) (691,279, Beyotime, Shanghai, China), total protein was recovered from the HGMCs on ice for 30 min. After being separated by 10% SDS-PAGE and transferred to polyvinylidene fluoride (PVDF) membranes (R8KA7385, Millipore, United States), equal amounts of proteins were used. Following 5% skim milk blocking, the membrane was incubated with the primary antibody for an overnight period at 4°C. The membrane was then incubated for 2 h at room temperature with secondary antibodies after being rinsed three times with PBST washing solution. The antibodies that were utilized are listed in the Supplementary Table S2 and the ratio of the target protein’s relative expression to β-actin, which served as the internal reference. Images were captured using an automatic exposure machine (JS-M6P, Shanghai Peiqing Technology Co., Ltd., China), and a luminescent film was prepared using an ECL high-sensitivity luminescence kit (S1257444, Thermo Scientific, United States). The relative protein expression was calculated by quantifying Western blot band intensities using the ImageJ analyzer software (version 1.48), and all tests were repeated three times.

### 2.7 Immunohistochemical staining

The clinical samples in this study were purchased from Chaohu Hospital of Anhui Medical University. The department of nephrology provided kidney biopsies from patients with CGN (n = 3), while the department of pathology provided samples of adjacent normal kidney tissue from patients with renal cell carcinoma (n = 3). This study’s ethical approval (KYXM-202208-006) was granted in accordance with the Declaration of Helsinki by Chaohu Hospital of Anhui Medical University. All patients signed informed permission forms after being informed of the purpose and significance of this study.

According to the manufacturer’s directions, a kidney specimen’s 3 m thick paraffin section was prepared for immunohistochemistry staining. At 37°C for 60 min, anti-FTO (1:50, 21j3698, affinity) and anti-FoxO6 (1:50, 78b1525, affinity) were incubated. All sections were incubated with the secondary antibody for 20 min at 37°C after being washed three times with 0.01 M PBS. The slices were then counterstained with hematoxylin (718034, Baso, Zhuhai, China) and stained with DAB (108038A08, Zs-BIO, Beijing, China). Sections were examined and captured on camera using a microscope (CX43, Olympus, Japan), and a brown product was obtained after positive staining. The average optical density (AOD) of FTO and FOXO6 was measured and analyzed using ImageJ v 1.51 software (NIH, United States).

### 2.8 EdU cell proliferation assay

Cell proliferation was assessed by adding EdU using the EdU-488 Click-iT Cell Proliferation Assay Kit (MPC2103010, Servicebio, Wuhan, China) per the manufacturer’s instructions. After incubating with EdU for 2 h, HGMCs were washed with PBS, rinsed with thymidine for 15 min, and fixed with 4% paraformaldehyde. EdU was introduced into proliferating cells by fluorescein-iF488 staining (Green) and nuclei were counterstained with Hoescht 33342 (Blue). Cell fluorescence images were taken using an inverted microscope (CKX53, Olympus, Japan), the rate of Edu-positive cell (%) was assessed by ImageJ v 1.51 software (NIH, United States), and all tests of each sample were analyzed in triplicate.

### 2.9 Detection of apoptosis using flow cytometry

Cell apoptosis was detected using the Apoptosis Kit (BB22061, Biosharp, Hefei, China) per the manufacturer’s instructions. Briefly, after trypsinization, precipitation, and washing twice with PBS, suspended cells were incubated with fluorescein isothiocyanate (46944, Sigma-Aldrich, Germany) and propylene iodide (81845, Sigma-Aldrich, Germany) for 15 min at room temperature in the dark. Cell apoptosis was detected using the flow cytometer (Novocyte, Agilent, United States) and analyzed with the flow cytometry software (NovoExpress 1.5.0, Agilent, United States). All tests of each sample were analyzed in triplicate.

### 2.10 RNA sequencing and data analysis

Illumina TruSeq RNA sample preparation v2 (Illumina, United States) was used to prepare RNA samples for sequencing on a next-generation sequencing platform. Following the manufacturer’s directions, total RNA was extracted from HGMCs using Redzol reagent (SBS Genetech, Beijing, China). FASTQC was used to evaluate read quality, and Seqtk (v1.3-r106) was used to filter it. After that, using HISAT2 with default settings, clean reads were mapped to the human reference genome version 38 (GRCh38/hg38). The expression of transcripts was then determined using the StringTie programme. The mRNAs with |Fold Change| ≥ 2 and *p*-value < 0.05 were considered significantly differentially expressed (Robinson et al., 2010; Sui et al., 2022).

### 2.11 Gene set download and processing

The gene sets of cell proliferation, apoptosis, and CGN were download from the GeneCard database (http://www.genecards.org), respectively. To increase the credibility of the gene set, the targets with relevance score ≥6.0 in cell proliferation and apoptosis gene set were screened (Hu et al., 2022). Finally, the cell proliferation gene set contained 9,817 genes, the cell apoptosis gene set contained 8,694 genes, the CGN gene set contained 2,031 genes, and all gene sets were used for subsequent analysis.

### 2.12 GO and KEGG enrichment analysis

The Gene Ontology (GO) and Kyoto Encyclopedia of Genes and Genomes (KEGG) analysis of related genes was performed using the bioinformatics website (https://www.bioinformatics.com.cn) (Luo and Brouwer, 2013), and a *p*-value *< 0.05* for the GO terms and KEGG pathways were considered statistically significant enriched.

### 2.13 Methylated RNA immunoprecipitation coupled with qPCR (MeRIP-qPCR)

Following to the manufacturer’s instructions, MeRIP-qPCR was carried out using the EpiQuik CUT&RUN m6A RNA Enrichment (MeRIP) Kit (2111015, Epigentek, United States). In a nutshell, HGMCs were treated with a TRIzol reagent (248207, Thermo Fisher, United States) to extract their total RNA. In order to do immunoprecipitation, RNA was fragmented and then treated with anti-m6A or anti-IgG antibodies. To guarantee that the RNA binding beads would successfully capture the RNA cloud, the RNA-antibody hybridization solution was incubated with the beads for 5 min at room temperature. The collected RNA was then released from the beads by suspending them in an elution solution at room temperature for 5 min. An RT-qPCR experiment was used to determine the enrichment of m6A-containing RNA after the supernatant was transferred to fresh RNase-free tubes. All tests of each sample were repeated three times.

### 2.14 RNA immunoprecipitation qPCR (RIP-qPCR)

RIP-qPCR assay was performed using the RNA Immunoprecipitation Kit (bes5101, BersinBio, Ghuangzhou, China) following the manufacturer’s instructions. Briefly, HGMCs were lysed in RIP lysis buffer and then immunoprecipitated with antibodies using protein A/G magnetic beads. HGMC lysate samples were incubated with either YTHDF3 antibody (IP) (0202730101, ABclonal, Wuhan, China) or control IgG antibody (IgG) at 4°C for 16 h. Finally, total RNA was extracted and analysed using RT-qPCR assay. All tests of each sample were repeated three times.

### 2.15 Actinomycin D assay

For the actinomycin D assay, HGMCs were treated with actinomycin D (M488106, Sigma, United States) for 0, 2, 4 and 8 h. Subsequently, RT-qPCR was used to detect FOXO6 mRNA expression levels, and all tests of each sample were repeated three times.

### 2.16 Statistical analysis

The statistical analysis was conducted using SPSS software (Version 22.0). One-way analysis of variance was used to compare the differences between the groups followed by the Tukey test, and a *p*-value lower than 0.05 was considered statistically significant. Plots were created using GraphPad Prism (version 8.0.2), with *p*-values indicated on the graph using ^*^ *p < 0.05* or ^**^ *p < 0.01* to denote statistical significance.

## 3 Results

### 3.1 Decreased expression of m6A demethylated transferase FTO in LPS-induced HGMCs

Before the start of the study, the optimal concentration and intervention time of LPS for subsequent cell experiments were determined using a CCK-8 assay. The CCK8 assay demonstrated that the viability of HGMCs was the strongest when the concentration of LPS was 1.0 μg/mL compared with control. Furthermore, no significant difference in cell viability was observed after stimulated with LPS (1.0 μg/mL) for 24 h and 48 h. Therefore, HGMCs stimulated with 1.0 μg/mL LPS for 24 h was selected for subsequent experiments (Supplementary Figure S1).

**FIGURE 1:**
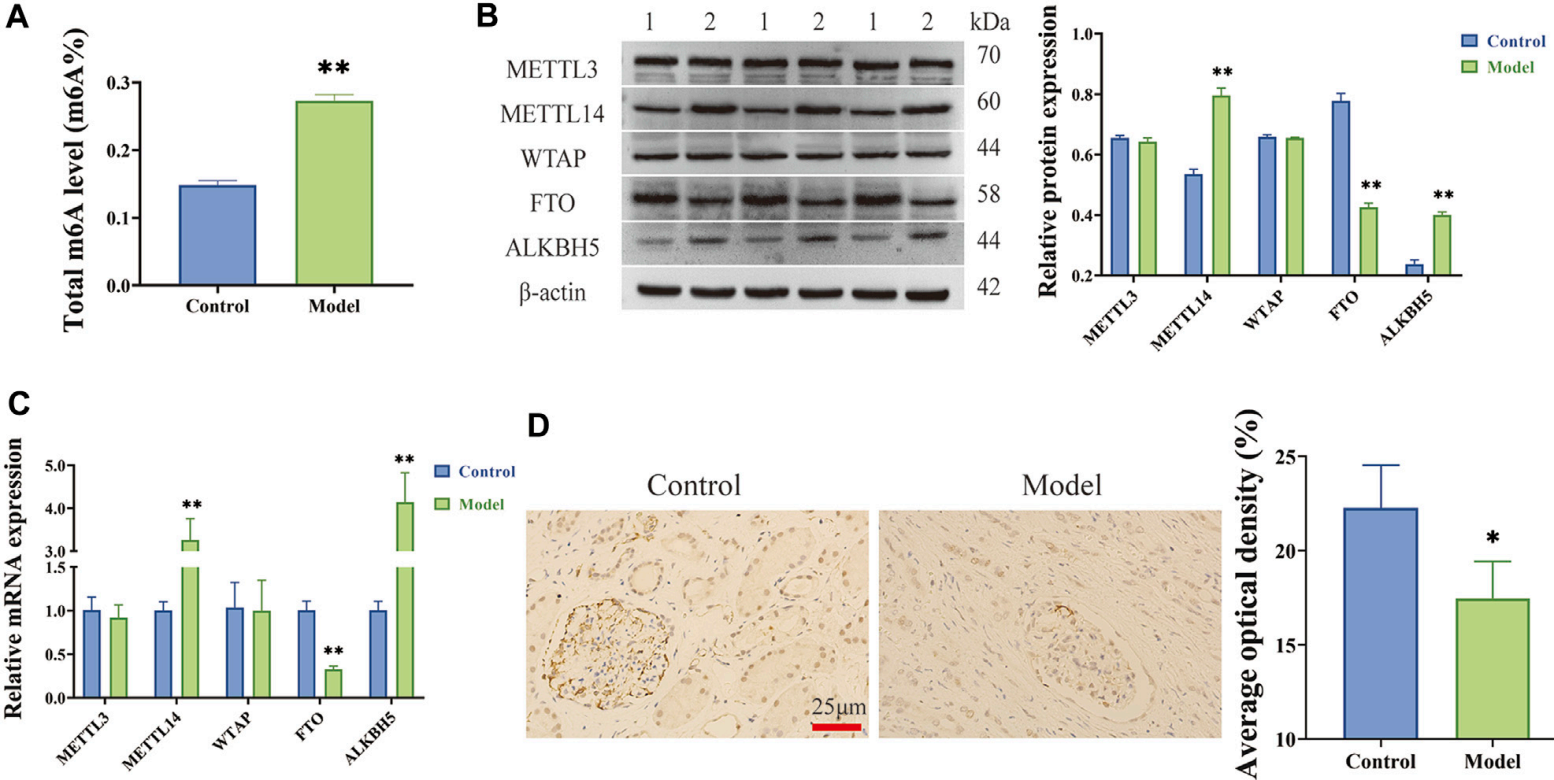
m6A modifications increased and m6A demethylated transferase FTO decreased in LPS-induced HGMCs. **(A)** Total m6A level increased in LPS-induced HGMCs; **(B)** The mRNA expression levels of METTL3, METTL14, WTAP, FTO and ALKBH5 detected by RT-qPCR; **(C)** The protein expression levels of METTL3, METTL14, WTAP, FTO and ALKBH5 detected by Western blot (1:Control; 2:Model); **(D)** The expression of FTO in glomerular units from patients with CGN detected by IHC staining assay (*n* = 3, ×400 magnification). ^*^ *p < 0.05*; ^**^ *p < 0.01*.

Using colourimetric analysis, the total level of m6A RNA modifications was found to be significantly elevated in LPS-induced HGMCs (Figure 1A). The mRNA and protein expression of methylated transferase (METTL3, METTL14, and WTAP) and demethylated transferase (FTO and ALKBH5) was then analysed using RT-qPCR and Western blot, respectively, revealing significantly increased METTL14 and ALKBH5 expression (*p < 0.01*) and significantly decreased FTO expression (*p < 0.01*) in LPS-induced HGMCs (Figures 1B, C). According to the characteristics of m6A methylases (Jiang et al., 2021a) and their associated *p* values, FTO was identified as a key m6A demethylated transferase in LPS-induced HGMCs. Furthermore, immunohistochemical analysis (Figure 1D) indicated that the expression of FTO in glomerular units from patients with CGN (Model) was significantly reduced compared with the adjacent normal kidney tissues (Control) (*p < 0.05*).

### 3.2 Regulatory effects of FTO overexpression or knockdown on proliferation and apoptosis of HGMCs

To investigate the regulatory effect of FTO on HGMC proliferation and apoptosis, we constructed FTO overexpression (OE-FTO) and knockdown (si-FTO) plasmids, respectively. The efficiency of FTO overexpression or knockdown was verified through Western blot, and the result was presented in Supplementary Figure S2.

**FIGURE 2:**
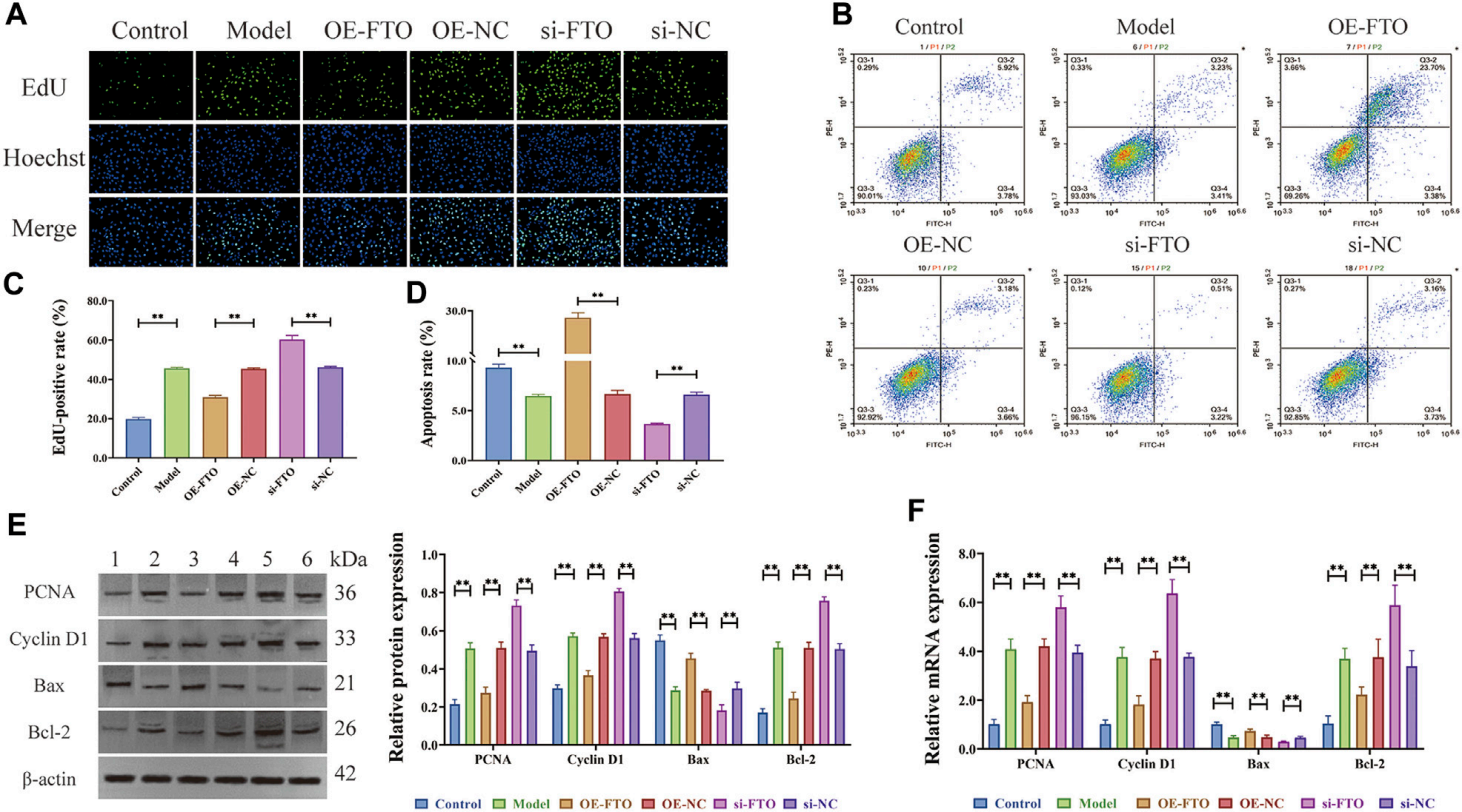
Regulatory effects of FTO overexpression and knockdown on proliferation and apoptosis of HGMCs. **(A, C)** Regulatory effects of FTO knockdown and overexpression on the proliferation of HGMCs detected by Edu cell proliferation assay (×400 magnification); **(B, D)** Regulatory effects of FTO knockdown and overexpression on the apoptosis of HGMCs detected by flow cytometry (Necrotic cells are represented by the first quadrant, late apoptotic cells by the second quadrant, normal cells by the third, and early apoptotic cells by the fourth. The ratio of cell apoptosis was represented by the relationship between the second and fourth quadrants.); **(E)** The protein levels of proliferation markers (Cyclin D1 and PCNA) and apoptosis markers (Bax and Bcl-2) detected by Western blot (1:Control; 2:Model; 3:OE-FTO; 4:OE-NC; 5:si-FTO; 6:si-NC); **(F)** The mRNA levels of proliferation markers (Cyclin D1 and PCNA) and apoptosis markers (Bax and Bcl-2) detected by RT-qPCR. ^*^ *p < 0.05*; ^**^ *p < 0.01*.

EdU assay (Figures 2A, C) revealed excessive proliferation of HGMCs in the model group compared to the control group (*p < 0.01*). Moreover, FTO overexpression significantly inhibited the excessive proliferation of HGMCs (*p < 0.01*), whereas FTO knockdown significantly promoted the excessive proliferation of HGMCs (*p < 0.01*). Flow cytometry (Figures 2B, D) revealed a decrease in the apoptosis rate of HGMCs in the model group compared to the control group (*p < 0.01*). FTO overexpression significantly promoted the apoptosis of HGMCs (*p < 0.01*), whereas FTO knockdown significantly the apoptosis of HGMCs (*p < 0.01*). The Western blot and RT-qPCR were employed for the detection of the protein and mRNA levels of proliferation markers (Cyclin D1 and PCNA) (Thatikonda et al., 2020) and apoptosis markers (Bax and Bcl-2) (Zhou et al., 2020). The results (Figures 2E, F) demonstrated that FTO overexpression significantly suppressed excessive proliferation and promoted the apoptosis of HGMCs (*p < 0.01*), whereas FTO knockdown significantly promoted the excessive proliferation and inhibited the apoptosis of HGMCs (*p < 0.01*).

### 3.3 Screening FOXO6 as the downstream target of FTO through RNA sequencing

**FIGURE 3:**
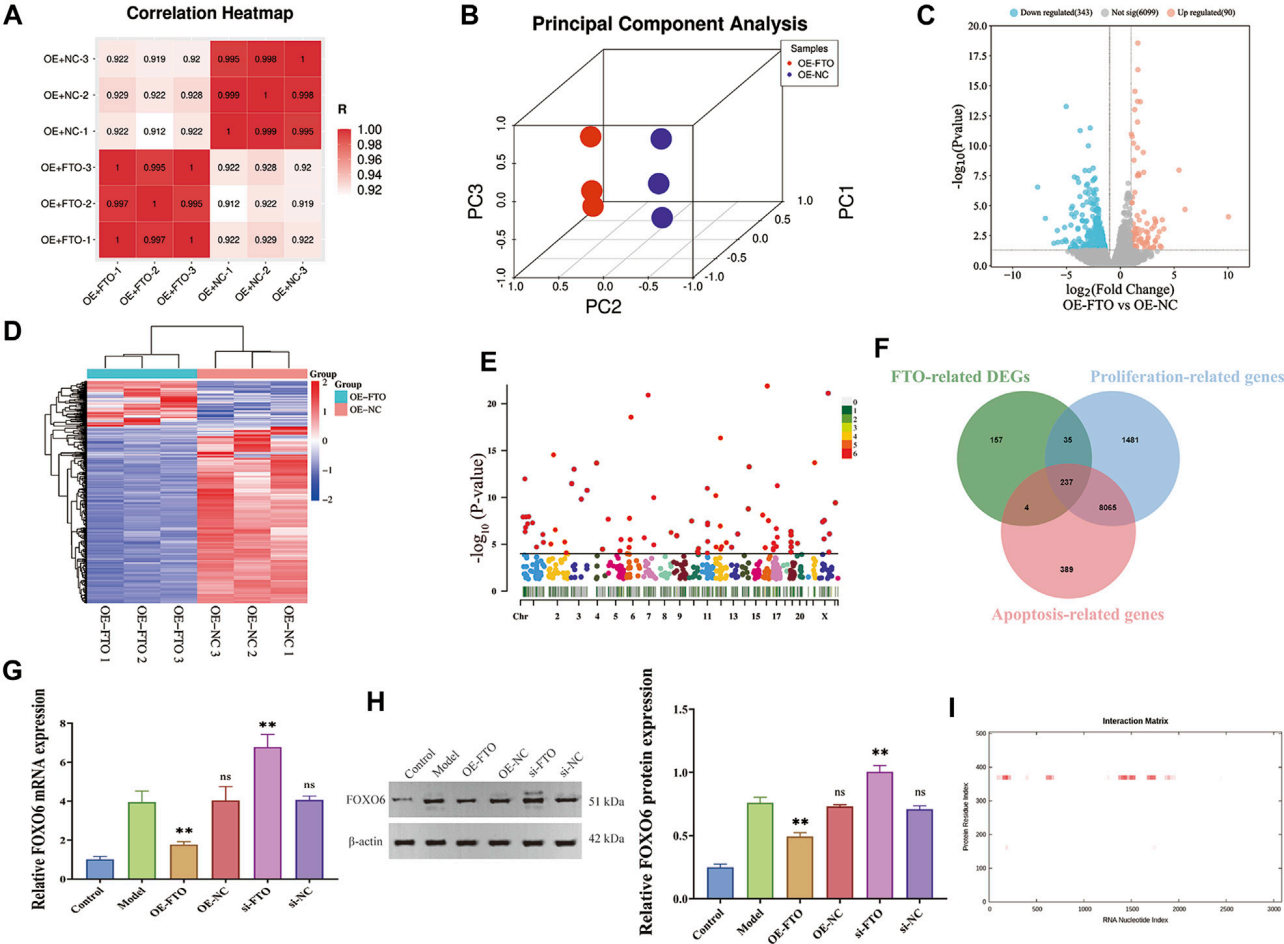
Screening FOXO6 as the downstream target of FTO through RNA sequencing. **(A)** The correlation heatmap between OE + FTO and OE + NC group; **(B)** The volcano map of DEGs; **(C)** Manhattan plot showing chromosomal distribution of DEGs; **(D)** The heatmap map of DEGs; **(E)** Effect of FTO knockdown and overexpression on FOXO6 mRNA expression detected by RT-PCR; **(F)** Venn diagram showing the intersection of FTO-related DEGs, proliferation-related genes, and apoptosis-related genes; **(G)** Effect of FTO knockdown and overexpression on FOXO6 mRNA expression detected by RT-qPCR; **(H)** Effect of FTO knockdown and overexpression on FOXO6 protein expression detected by Western blot; **(I)** The interaction map of FTO protein and FOXO6 mRNA. Compared with the model group, ^*^ *p < 0.05*; ^**^ *p < 0.01*.

To explore the downstream target of FTO, differentially expressed genes (DEGs) between FTO overexpression + LPS-induced HGMCs (OE + FTO) and NC overexpression + LPS-induced HGMCs (OE + NC) were identified using RNA sequencing. The quality control of the RNA samples, sequencing libraries and sequencing reads are presented in Supplementary Table S3. Correlation analysis of the samples indicated good repeatability within the sample group and significant differences between groups, as shown in the correlation heatmap (Figure 3A). Principal component analysis (PCA) plots revealed significant overall differences among the groups (Figure 3B). A total of 6,532 mRNAs were identified using RNA sequencing, with 433 mRNAs (Fold Change ≥ 2.0 and *p < 0.05*) considered as DEGs, comprising 343 downregulated mRNAs and 90 upregulated mRNAs. The volcano map and heatmap of DEGs are shown in Figures 3C, D. The Manhattan plot (Figure 3E) showed that DEGs were mainly distributed on chromosomes 1, 17, 12, 2 and 11.

We intersected the FTO-related DEGs with the gene set of CGN, and 50 intersection genes were finally obtained (Supplementary Figure S3A). Then, Kyoto Encyclopedia of Genes and Genomes (KEGG) analysis was performed on the 50 intersection genes. A total of 23 signaling pathways were significantly enriched by KEGG analysis, including Growth hormone synthesis, secretion and action, FOXO signaling pathway, ECM-receptor interaction, TNF signaling pathway, Cell cycle, PI3K-AKT signaling pathway, JAK-STAT signaling pathway, *etc.* (*p < 0.05*, Supplementary Figure S3B and Supplementary Table S4).

To find the downstream genes of FTO in regulating proliferation and apoptosis of HGMCs, we further intersected FTO-related DEGs with the gene sets of cell proliferation and apoptosis, and finally obtained 237 mRNAs (Figure 3F). Considering the expression level, fold change and the *p-*value, Forkhead Box O6 (FOXO6) wtih log_2_FC = −3.5408, and *p* = 1.7450 × 10^−4^ was identified as an important downstream gene of FTO. To verify the upstream and downstream relationship between FTO and FOXO6, RT-qPCR and Western blot were performed to detect FOXO6 expression in the presence of FTO overexpression or knockdown. The RT-qPCR and Western blot analysis results (Figure 3G; Figure 3H) revealed that FOXO6 was significantly increased in LPS-induced HGMCs (*p < 0.01*), whereas FTO overexpression decreased FOXO6 mRNA expression (*p < 0.01*). Meanwhile FTO knockdown increased the mRNA expression of FOXO6 (*p < 0.01*). Immunohistochemical analysis (Figure 3H) indicated a significant elevation of FOXO6 expression in kidney tissues from patients with CGN compared to adjacent normal kidney tissues (Supplementary Figure S4).

Through the catRAPID website (http://s.tartaglialab.com/page/catrapid_group) (Livi et al., 2016; Armaos et al., 2021), we discovered that the FTO protein can bind to multiple regions of FOXO6 mRNA, and the interaction map was presented in Figure 3I. Results of prediction from catRAPID website showed that the 355–406 region of FTO protein can bind to multiple regions of FOXO6 mRNA, and the specific binding regions were listed in Table 1. Taken together, these findings suggest that FOXO6 is a downstream target of FTO, and its expression in CGN is negatively correlated with FTO.

**TABLE 1:** Binding region of FTO protein and FOXO6 mRNA predicted by catRAPID website.

NO.	Protein region	RNA region	Interaction propensity	Discriminative power	Normalized score
1	355–406	158–281	54.99	96	3.53
2	355–406	1,404–1,527	54.28	96	3.46
3	355–406	1,465–1,588	53.97	96	3.43
4	355–406	1,683–1806	53.67	96	3.40
5	355–406	1,378–1,501	52.98	95	3.34
6	355–406	123–246	52.85	95	3.33
7	355–406	1,709–1,832	52.52	95	3.29
8	355–406	1,500–1,623	52.19	95	3.26
9	355–406	611–734	51.48	95	3.20
10	355–406	1,648–1771	51.12	95	3.16
11	355–406	585–708	50.54	95	3.11
12	355–406	1,343–1,466	49.94	94	3.05
13	355–406	184–307	48.84	94	2.95
14	355–406	1,439–1,562	48.72	94	2.93
15	355–406	1,866–1,989	48.43	94	2.91
16	355–406	646–769	47.17	93	2.79
17	355–406	62–185	46.38	92	2.71
18	355–406	1,831–1,954	46.32	92	2.71
19	355–406	1,892–2,015	45.86	92	2.66
20	355–406	97–220	45.78	92	2.66

### 3.4 FOXO6 is a downstream target of FTO in regulating the proliferation and apoptosis of HGMCs

**FIGURE 4:**
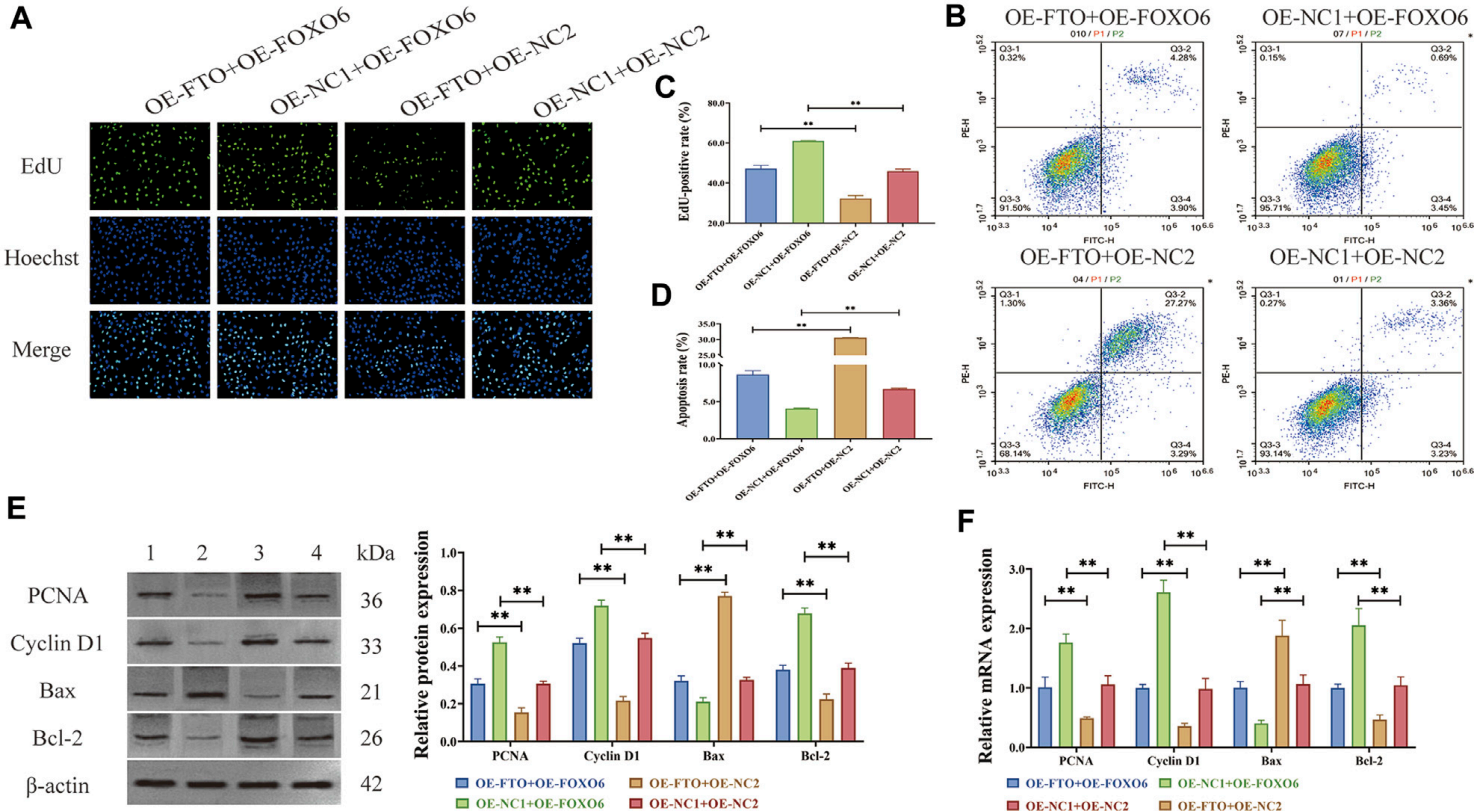
FOXO6 is a downstream target of FTO in regulating proliferation and apoptosis of HGMCs. **(A, C)** Effect of FOXO6 overexpression on the proliferation of HGMCs detected by Edu cell proliferation assay under the condition of FTO overexpression (×400 magnification); **(B, D)** Effect of FOXO6 overexpression on the apoptosis of HGMCs detected by flow cytometry under the condition of FTO overexpression (Necrotic cells are represented by the first quadrant, late apoptotic cells by the second quadrant, normal cells by the third, and early apoptotic cells by the fourth. The ratio of cell apoptosis was represented by the relationship between the second and fourth quadrants.); **(E)** The protein levels of proliferation markers (Cyclin D1 and PCNA) and apoptosis markers (Bax and Bcl-2) detected by Western blot (1:OE-FTO + OE-FOXO6; 2:OE-NC1+OE-FOXO6; 3:OE-FTO + OE-NC2; 4:OE-NC1+OE-NC2); **(F)** The mRNA levels of proliferation markers (Cyclin D1 and PCNA) and apoptosis markers (Bax and Bcl-2) detected by RT-qPCR. ^*^ *p < 0.05*; ^**^ *p < 0.01*.

As a downstream target of FTO, FOXO6 has been confirmed in literature reports to regulate cell proliferation and apoptosis (Abdalla et al., 2021; Tang et al., 2021). However, further investigation is needed to determine whether FTO affects the proliferation and apoptosis of HGMCs by regulating FOXO6. To address this, FTO overexpression plasmids (OE-FTO, OE-NC1) and FOXO6 overexpression plasmids (OE-FOXO6, OE-NC2), as well as controls, were transfected into LPS-induced HGMCs. EdU assay (Figures 4A, C) revealed that FOXO6 overexpression can significantly counteract the inhibitory effect of FTO overexpression on the excessive proliferation of HGMCs (*p < 0.01*). Meanwhile, flow cytometry detection of apoptosis (Figures 4B, D) showed that FOXO6 overexpression significantly counteracted the promoting effect of FTO overexpression on apoptosis of HGMCs (*p < 0.01*). Consistent with these findings, Western blot and RT-qPCR (Figures 4E, F) confirmed that the expression levels of proliferation markers (Cyclin D1 and PCNA) and apoptosis markers (Bax and Bcl-2) were consistent with the observed effects on proliferation and apoptosis. In conclusion, these findings indicate that FOXO6 is a downstream target of FTO in the regulation of proliferation and apoptosis of HGMCs.

### 3.5 FTO regulates FOXO6 m6A modification via YTHDF3-dependent manner

Previous experiments showed that FOXO6 is a downstream target of FTO in the regulation of proliferation and apoptosis of HGMCs. However, the exact mechanism through which FTO regulates FOXO6 expression remains to be elucidated. According to the m6A2Target website (http://rm2target.canceromics.org), a comprehensive database containing m6A writer, eraser, and reader target genes, FTO could regulate the m6A modification of FOXO6 (Table 2). Given this, we hypothesized that FTO, being an m6A demethylated transferase, may regulate the expression of FOXO6 through m6A modification (Jiang et al., 2021b; Tao et al., 2021).

**TABLE 2:** The binding relationship between FTO and FOXO6 predicted by RM2Target website.

RM2Target ID	WERs name	WERs type	Target gene	Modification	Organism	Cell line/Tissue
RM2Target_707061	FTO	Eraser	FOXO6	m6A	*Homo sapiens*	OVCAR5
RM2Target_1598160	FTO	Eraser	FOXO6	m6A	*Mus musculus*	hippocampus

**FIGURE 5:**
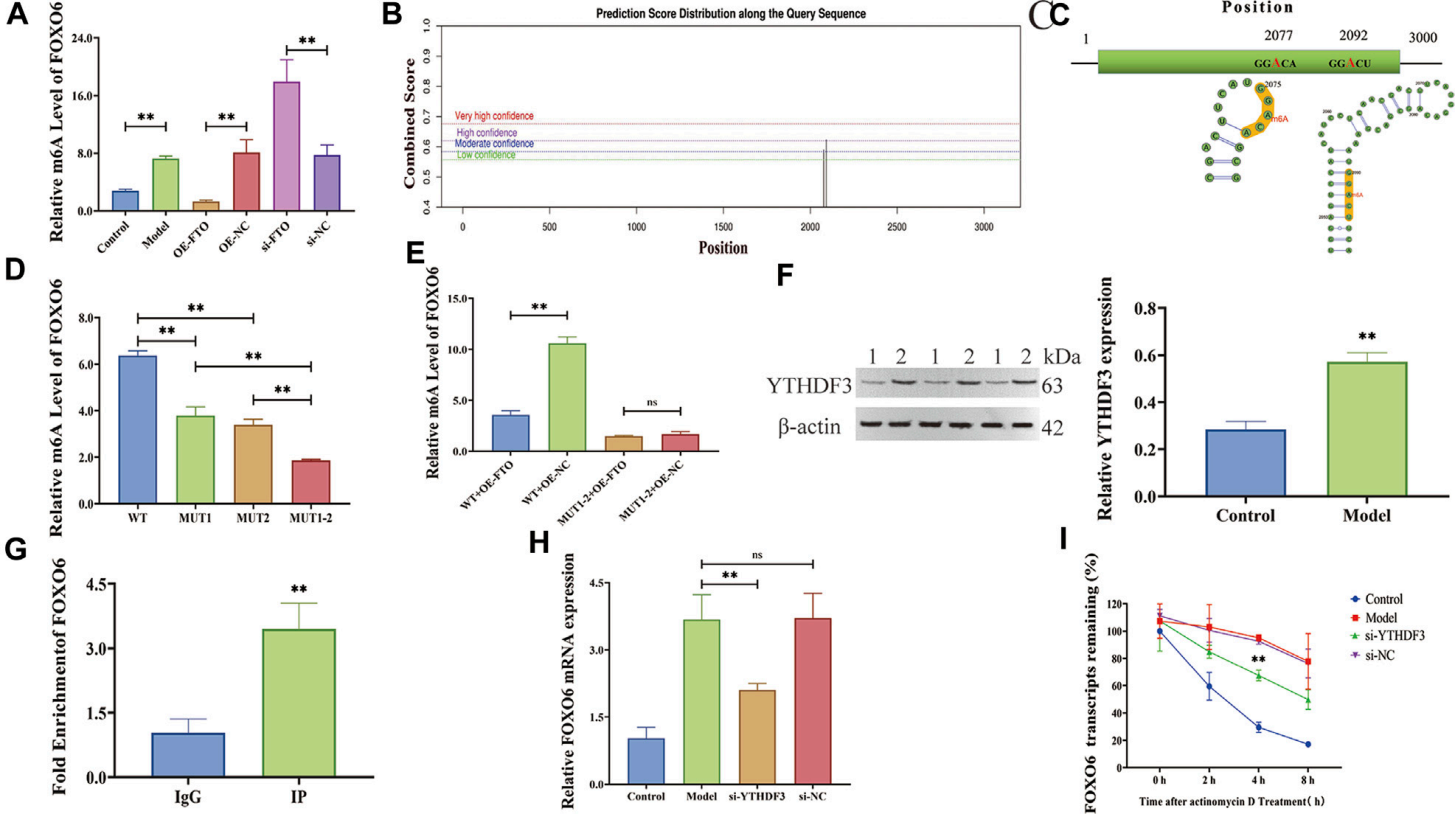
FTO regulates FOXO6 m6A modification via YTHDF3-dependent manner. **(A)** Effect of FTO overexpression and knockdown on m6A level of FOXO6 detected by MeRIP-qPCR; **(B)** The potential m6A sites in FOXO6 predicted by SRAMP website; **(C)** The secondary RNA structure and location of m6A site on FOXO6 mRNA; **(D)** The m6A level of FOXO6 detected by MeRIP-qPCR after methylation site mutation; **(E)** Effect of FTO overexpression on m6A level of FOXO6 detected by MeRIP-qPCR after methylation site mutation; **(F)** The expression level of m6A recognition protein YTHDF3 detected by Western blot (1:Control; 2:Model); **(G)** RIP‐qPCR detection of the binding relationship between YTHDF3 and FOXO6; **(H)** The effect of YTHDF3 knockdown on FOXO6 expression detected by RT-qPCR; **(I)** The effect of YTHDF3 knockdown on FOXO6 mRNA stability detected by actinomycin D assay. ^*^ *p < 0.05*; ^**^ *p < 0.01*.

To confirm the above hypothesis, m6A level of FOXO6 was determined using a MeRIP-qPCR assay, which revealed that FTO overexpression or knockdown significantly reduced or increased the m6A modification level of FOXO6 (Figure 5A) (*p < 0.01*). The potential m6A sites in FOXO6 were predicted using the SRAMP website, revealing two methylation sites (2077 and 2092 base position) in FOXO6 mRNA (Figure 5B and Table 3). The secondary RNA structure and location of m6A modification sites in FOXO6 was shown in Figure 5C. To confirm the m6A modification site in FOXO6 mRNA, we mutated the methylated sites (MUT1:2077; MUT2:2092; MUT1-2:2077 and 2092). After the mutation, the m6A level of FOXO6 was significantly reduced in the MUT1 and MUT2 groups (*p < 0.01*), compared with the wild-type (WT) group. Moreover, the m6A level of FOXO6 in MUT1-2 was lower than that in the MUT1 and MUT2 groups (*p < 0.01*), which indicated that the m6A modification exists at the 2077 and 2092 methylation sites (Figure 5D). After mutating the two methylated sites, FTO overexpression had no effect on the m6A level of FOXO6, suggesting that FTO affects the m6A level of FOXO6 through the 2077 and 2092 methylation sites (Figure 5E).

**TABLE 3:** The potential m6A sites in FOXO6 predicted by SRAMP website.

Position	Sequence context	Score (binary)	Score (knn)	Score (spectrum)	Score (combined)	Decision
2077	AUCAU CCUCA ACGAC	0.611	0.754	0.542	0.590	Moderate confidence
	UUCAU **GGACA** GCGAC					
	GAAAU GGACU UCAAC					
2092	UUCAU GGACA GCGAC	0.697	0.530	0.536	0.624	High confidence
	GAAAU **GGACU** UCAAC					
	UUCGA UUCGG CCCUG					

YTH domain family 3 (YTHDF3), an m6A reader, was discovered to bind to FOXO6 according to the m6A2Target website (Table 4). Given that YTHDF3 is known to play a crucial role in regulating the stability and translation of m6A-modified mRNAs (Sun et al., 2022a), we investigated the potential contribution of YTHDF3 in the stabilization of FOXO6 mRNA.The expression level of YTHDF3 protein was detected by Western blot revealing a significant increase in YTHDF3 expression in the LPS-induced HGMCs (*p < 0.01*) (Figure 5F). RIP-qPCR assay showed that the YTHDF3 specific antibody (IP) was significantly enriched with FOXO6 mRNA, compared with the IgG control antibody (*p < 0.01*, Figure 5G). YTHDF3 knockdown significantly reduced the expression of FOXO6 mRNA (*p < 0.01*, Figure 5H). Moreover, the actinomycin D experiment showed that the stability of FOXO6 mRNA decreased after YTHDF3 knockdown (*p < 0.01*, Figure 5I), indicating that YTHDF3 plays a critical role in the maintenance of FOXO6 mRNA stability.

**TABLE 4:** The binding relationship between YTHDF3 and FOXO6 predicted by RM2Target website.

RM2Target ID	WERs name	WERs type	Target gene	Modification	Organism	Cell line/Tissue
RM2Target_1237319	YTHDF3	Reader	FOXO6	m6A	*Homo sapiens*	HeLa

Taken together, these findings suggested that FTO can regulate the m6A modification of FOXO6 mRNA, and influence the stability of FOXO6 mRNA via YTHDF3-dependent manner, consequently impacting the expression of FOXO6 mRNA.

### 3.6 FTO overexpression and FOXO6 knockdown could inhibit the PI3K/AKT signaling pathway

**FIGURE 6:**
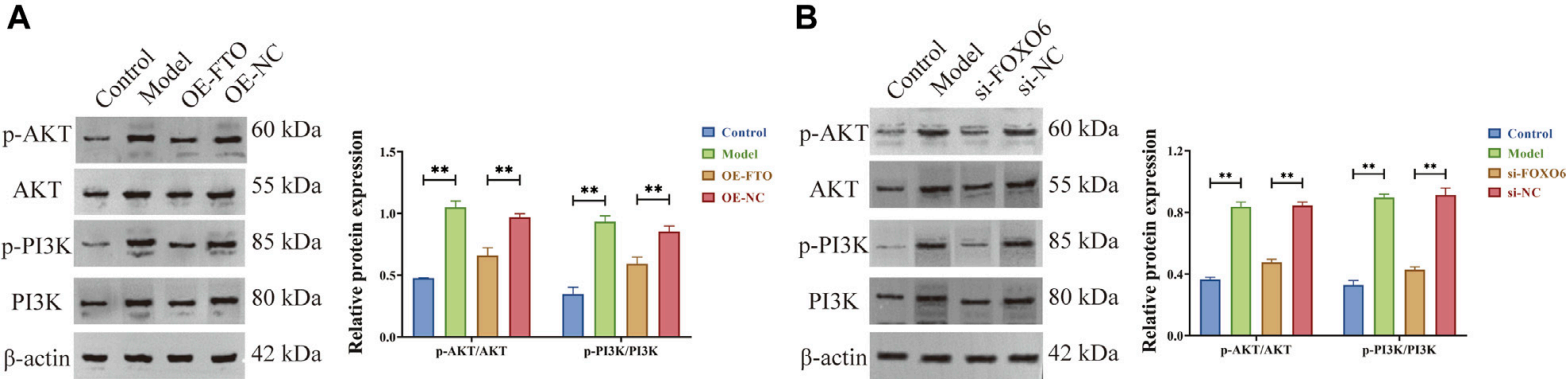
FTO overexpression and FOXO6 knockdown could inhibit the PI3K/AKT signaling pathway. **(A)** Effect of FTO overexpression on PI3K/AKT signaling pathway; **(B)** Effect of FOXO6 knockdown on PI3K/AKT signaling pathway.

According to literature reports, FOXO6 can activate the phosphoinositide 3-kinase (PI3K)/serine-threonine kinase (AKT) signaling pathway, thereby promoting cell proliferation and inhibiting apoptosis (Feng and Qiu, 2018; Wang et al., 2019; Wu et al., 2020). Meanwhile, KEGG enrichment analysis suggested that FTO is involved in the pathogenesis of CGN through PI3K/AKT signaling pathway (Supplementary Figure S3B and Supplementary Table S4). Herein, Western blot results (Figure 6A and Figure 6B) revealed that FTO overexpression and FOXO6 knockdown could significantly inhibit AKT and PI3K phosphorylation, thus inhibiting the PI3K/AKT signaling pathway.

Based on the above experimental findings in this manuscript and literature reports, it can be concluded that FTO overexpression could inhibit the PI3K/AKT signaling pathway by suppressing the expression of FOXO6, thereby inhibiting excessive proliferation and promoting apoptosis of HGMCs.

## 4 Discussion

**FIGURE 7:**
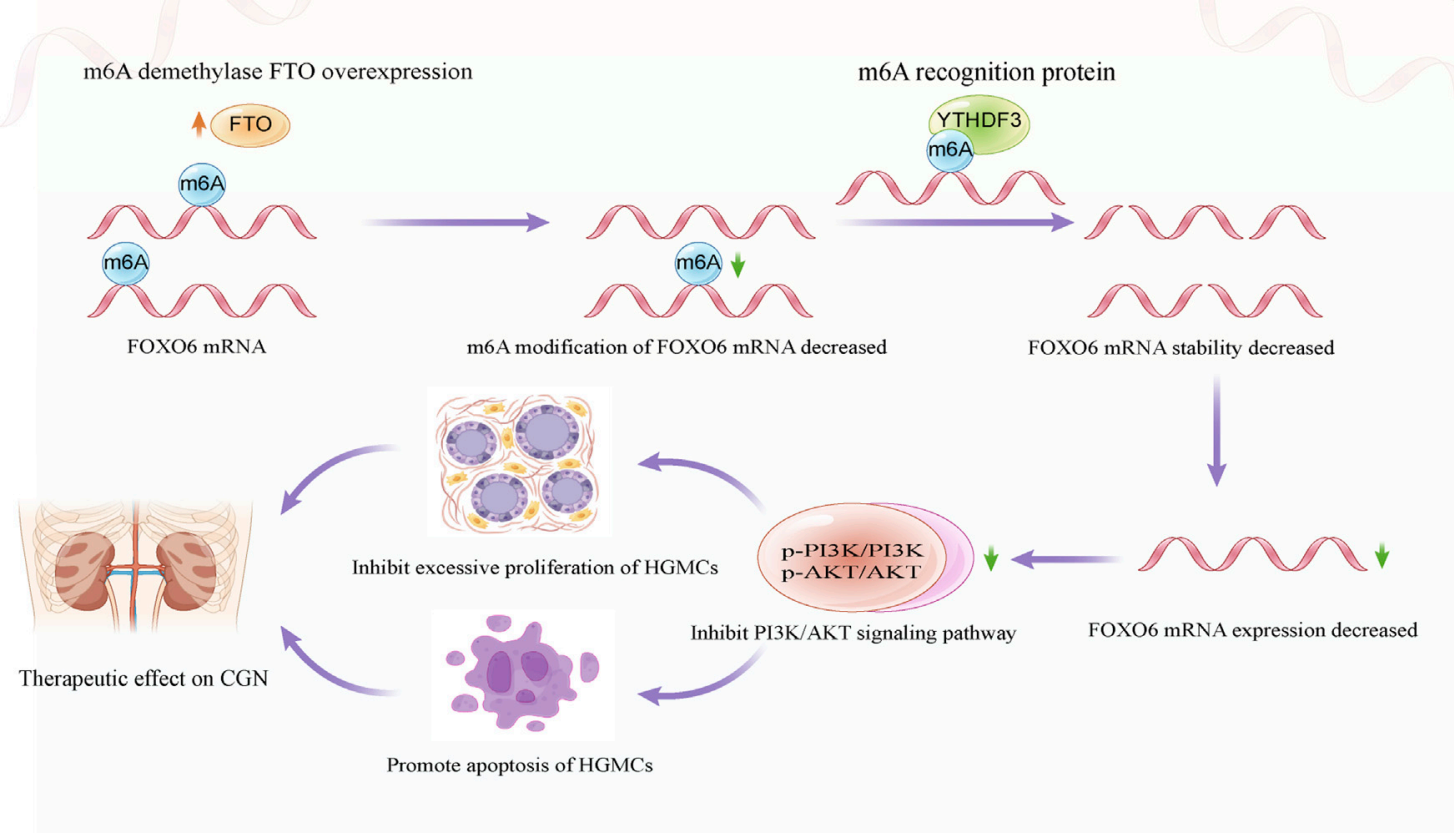
Schematic diagram depicting the molecular mechanism of FTO regulating the proliferation and apoptosis of HGMCs.

In the present study, FTO, the m6A demethylated transferase, was found significantly reduced in LPS-induced HGMCs and glomerular units obtained from patients with CGN. Additionally, RNA sequencing and cellular experiments revealed FOXO6 as a downstream target of FTO in regulating the proliferation and apoptosis of HGMCs. Mechanistically, FTO overexpression decreases the level of FOXO6 m6A modification and reduces the stability of FOXO6 mRNA in a YTHDF3-dependent manner. Additionally, the decreased expression of FOXO6 inhibits the PI3K/AKT signaling pathway, thereby inhibiting the proliferation and promoting apoptosis of HGMCs. The schematic diagram depicting the proposed mechanism is shown in Figure 7. These findings highlight the role of m6A modification in the pathogenesis of CGN and suggest FTO as a potential diagnostic marker and therapeutic target in CGN.

FTO, originally known for its role in regulating metabolism and energy utilisation, has been closely associated with the regulation of body fat mass and the risk of obesity (Zhao et al., 2014). Recent studies have confirmed that FTO is closely related to m6A modification. Notably, FTO can preferentially catalyse m6A demethylation modification in a ferrous ion (Fe 2+)/2-oxoglutarate (2-OG)-dependent manner. It can also regulate post-transcriptional downstream target mRNA stability, RNA processing, splicing, localisation, translocation, monitoring, decay and translation through m6A modification (Yang et al., 2021; Azzam et al., 2022). For example, Xu et al. reported that FTO-mediated m6A modification regulates SIK2, a functional target of m6A-mediated autophagy, expression through IGF2BP2-dependent SIK2 mRNA stability, thereby promoting renal cell carcinoma (Xu et al., 2022). Sun et al. revealed a protective role of FTO during diabetic kidney disease (DKD) pathogenesis. FTO expression is significantly decreased in DKD, and its overexpression can alleviate kidney inflammation by modulating them6A modification of SOCS1 (Sun et al., 2022b). Additionally, Yang et al. revealed that the reduced expression of FTO stabilised SQSTM1 mRNA by increasing its m6A modification levels, leading to the formation of autophagosomes and the promotion of renal fibrosis (Yang et al., 2023). In the present study, we observed that the expression of FTO was significantly reduced in LPS-induced HGMCs and glomerular units of patients with CGN. FTO overexpression could inhibit excessive proliferation and promote apoptosis of HGMCs by alleviating FOXO6 m6A modification through YTHDF3-dependent mechanisms in CGN. These findings highlight the crucial role of FTO-mediated m6A modification in kidney diseases and indicate the potential of FTO as an important marker and therapeutic target for kidney diseases.

Cell proliferation refers to the division and reproduction of cells to produce new cells, and apoptosis is the programmed cell death to ensure cellular homeostasis. In multicellular organisms, cell proliferation and apoptosis are important fundamental processes in the cellular life cycle, and their balance is crucial to cell development and tissue health (Saleh et al., 2000; Wani et al., 2021). The regulatory mechanism of cell proliferation and apoptosis is complex, involving various signalling pathways and molecular regulators. Under normal physiological conditions, GMCs maintain a dynamic balance between proliferation and apoptosis. When stimulated by physiological or external pathological factors, GMCs are activated, leading to excessive proliferation and inadequate apoptosis. However, excessive GMC proliferation results in mesangial expansion, increased glomerular extracellular matrix deposition in the mesangial region and ultimately decreased glomerular filtration rate (Scindia et al., 2010; Pereira et al., 2021). Therefore, inhibiting excessive GMC proliferation and promoting proper GMC apoptosis are vital strategies for controlling the progression of CGN in its initial stages.

The relationship between FOXO6 and PI3K/AKT signalling pathway has been established in various literature reports. For example, Yu’s study showed that depletion of FOXO6 can inhibit glycolysis in HCC cells through the PI3K/AKT pathway and reduce the resistance of cells to paclitaxel (Yu et al., 2020). Additionally, Li’s research found that knockdown of FOXO6 could inhibit the phosphorylation of PI3K, AKT and mTOR (*p < 0.01*), and decrease the expressions of P-PI3K, P-AKT and P-mTOR in colorectal cancer cells. The research indicated that FOXO6 knockdown inhibited cell proliferation, migration, invasion and glycolysis of colorectal cancer cells by inhibiting the PI3K/AKT/mTOR signaling pathway (Li et al., 2019). Notably, the PI3K/AKT signaling pathway is the most common signaling pathway associated with cell proliferation and apoptosis. Studies by Feng et al. showed that artesunate inhibited the proliferation of chondrocytes in RA rats and promoted apoptosis and autophagy by inhibiting the PI3K/AKT/mTOR signaling pathway (Feng and Qiu, 2018). Similarly, Chai et al. reported that microRNA-21 promotes human glioma cell proliferation and inhibits cell senescence and apoptosis by targeting SPRY1 through PTEN/PI3K/AKT pathway (Chai et al., 2018). Additionally, Bai et al. found that ibuprofen could inhibit fibrosarcoma cell proliferation, cell cycle and apoptosis through PI3K/AKT/mTOR signaling pathway (Bai et al., 2022). The current study demonstrated that FOXO6, as a downstream target of FTO, could regulate the proliferation and apoptosis of HGMCs. Moreover, further research showed that FOXO6 knockdown could inhibit proliferation and promote apoptosis of HGMCs by inhibiting the PI3K/AKT signaling pathway. Overall, our findings are consistent with that of previous studies.

This study delineated the molecular mechanism by which FTO regulates the proliferation and apoptosis of HGMCs by mediating FOXO6 m6A modification at the cellular level *in vitro*. As an *in vitro* cellular mechanism study, we should acknowledge that there are several limitations of our study. First of all, the relationship between FTO and the clinical features of CGN, and the vital role of FTO in the pathogenesis of CGN need to be further explored. Second, the m6A regulatory relationship between FTO and FOXO6 need to be confirmed by *in vivo* experiments. Nevertheless, as the first report of FTO-mediated m6A modification in CGN, this study provides a valuable insight and reference for future research on m6A modification in CGN.

## Data Availability

The datasets presented in this study can be found in online repositories. The names of the repository/repositories and accession number(s) can be found below: NCBI database (BioProject ID: PRJNA1017635).
