# Supplementary material for: Overexpression of FTO inhibits excessive proliferation and promotes the apoptosis of human glomerular mesangial cells by alleviating FOXO6 m6A modification via YTHDF3-dependent mechanisms
Source: Front Pharmacol. 2023 Sep 26;14:1260300. doi: 10.3389/fphar.2023.1260300 (PMC10562590; doi:10.3389/fphar.2023.1260300)
Supplement: Supplementary file 1 [file DataSheet1.DOC]

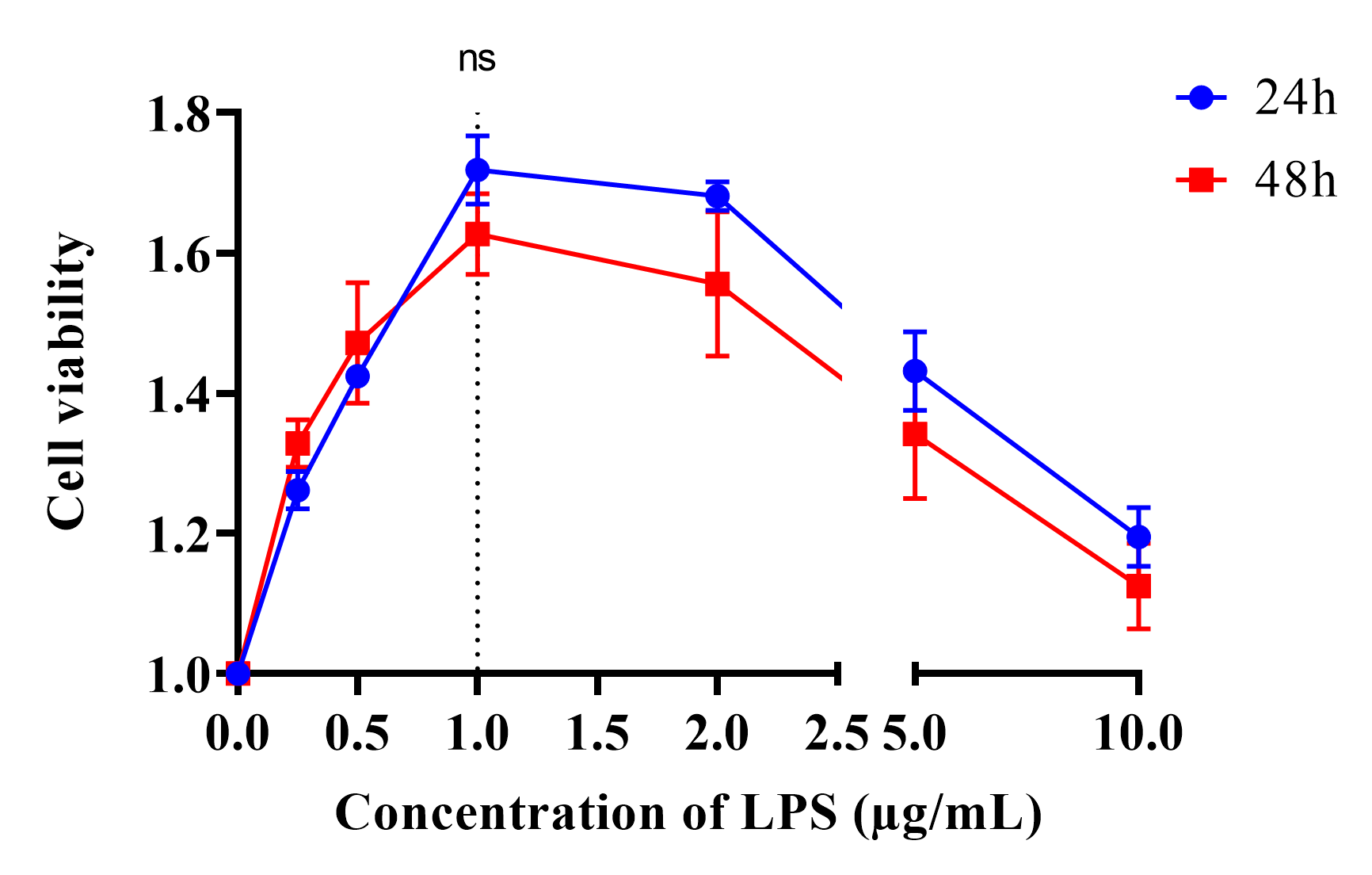


**Figure S1. Concentration and time screening of LPS-induced HGMCs by CCK8 assay.**


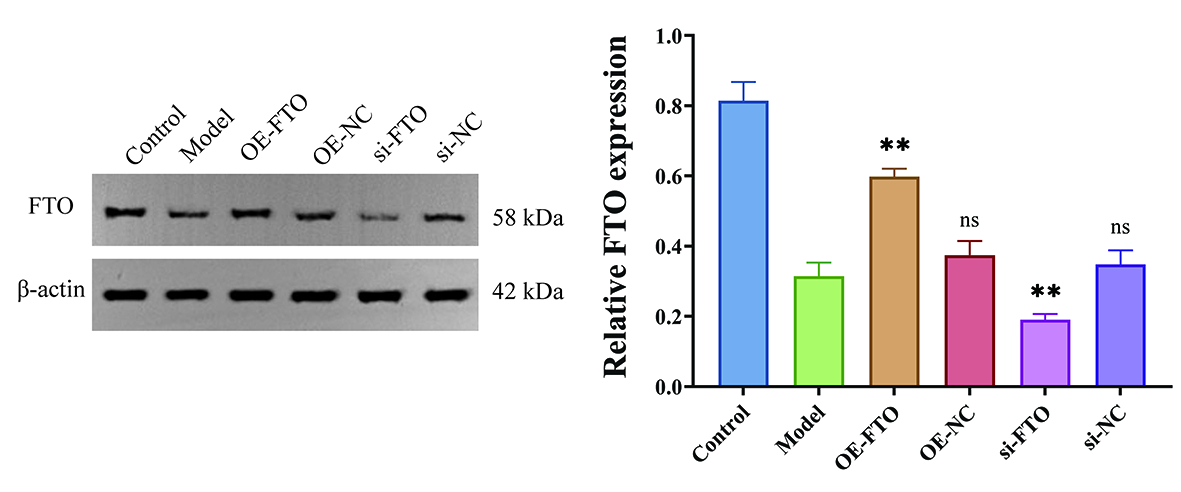


**Figure S2. The efficiency of FTO overexpression and knockdown verified by western blot.** Compared with the model group, ^*^*P < 0.05*; ^**^*P < 0.01*.


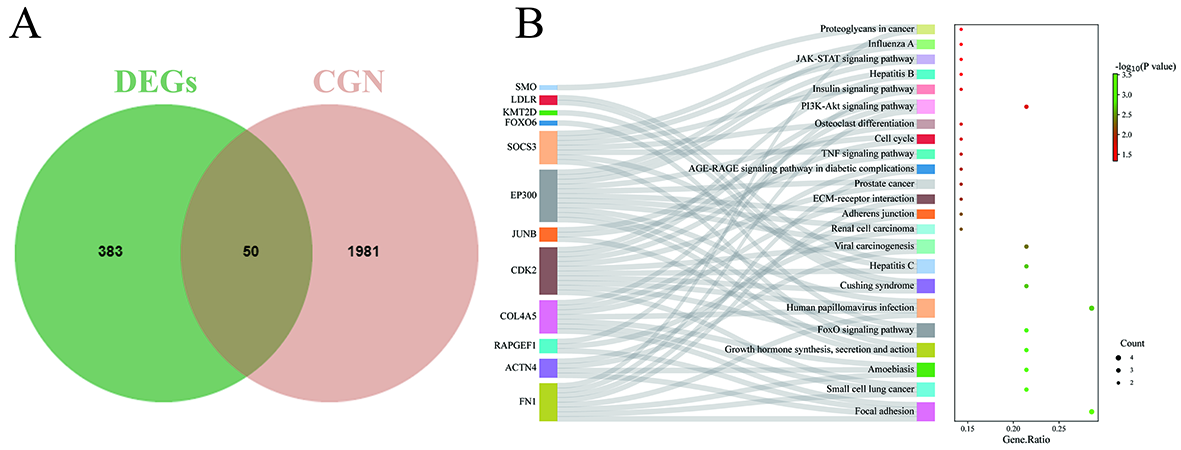


**Figure S3. KEGG analysis of FTO-related DEGs involved in CGN.** A, Intersection of FTO-related DEGs and CGN-related genes. B, All significantly enriched signaling pathways in KEGG analysis of FTO-related DEGs involved in CGN.


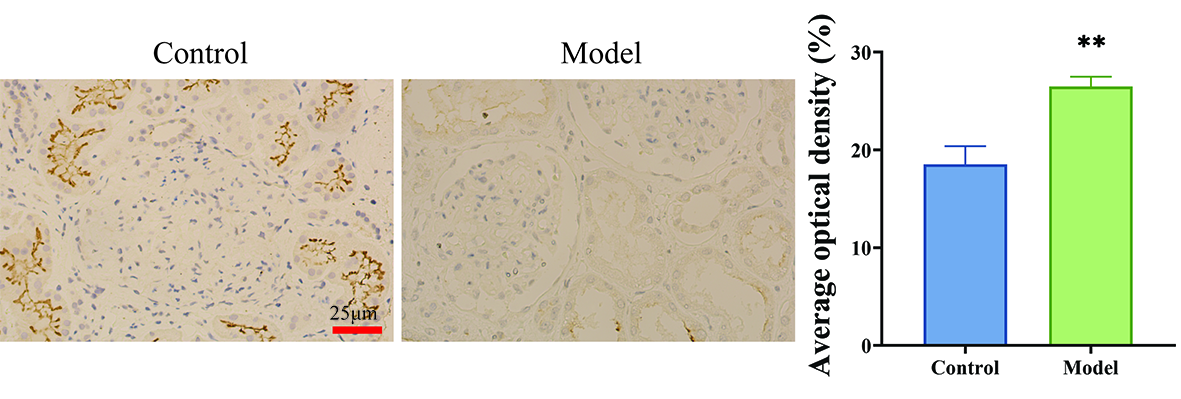


**Figure S4. The expression of FOXO6 in glomerular units from patients with CGN detected by IHC staining assay** (n=3, × 400 magnification). ^*^*P < 0.05*; ^**^*P < 0.01*.

**Table S1 The primers used for RT-qPCR analysis**

| Gene | Amplicon Size  （bp） | Forward primer  （5'→3'） | Reverse primer  （5'→3'） |
| --- | --- | --- | --- |
| METTL3  METTL14  WTAP  FTO  ALKBH5  PCNA  Cyclin D1  Bax  Bcl-2  FOXO6  β-actin | 184  132  163  148  96  169  93  103  94  102  96 | GCCTTCTGAACCAACAGTCC  TGCCTGTGATGGGTCCTTAG  AAGCAACAACAGCAGGAGTC  AGACACCTGGTTTGGCGATA  TCTGCACTTGGTTGAGGTCT  AGCCGAAACCAGCTAGACTT  ATGAACTACCTGGACCGCTT  GCCTCCTCTCCTACTTTGGG  TCTGCGAATACCGGACTGAA  CTCGACATGTTCAGCGGGAG  CCCTGGAGAAGAGCTACGAG | CTGGCTTTCATGCACTCCTC  ACAGGTGCCTATGCCATGTA  TCGCTGGGTCTACCATTGTT  GTTCCTGTTGAGCACTCTGC  AGGGTGTTTGCATGAGCTTG  ACCGCTGGAGCTAATATCCC  CTTAGAGGCCACGAACATGC  CCTCAGCCCATCTTCTTCCA  CAGGAATCCCAACCGGAGAT  CAGGGCCGAATCGAAGTTGA  GGAAGGAAGGCTGGAAGAGT |

**Table S2 The antibodies information for western blot**

| Product name | Product brand | Batch No. | Molecular Weight | Antibody Source | Dilution Ratio | Separating Gel Concentration |
| --- | --- | --- | --- | --- | --- | --- |
| METTL3  METTL14  WTAP  FTO  ALKBH5  Cyclin D1  PCNA  Bax  Bcl-2  AKT  P-AKT  PI3K  P-PI3K  β-actin  goat anti-mouse IgG  goat anti-rabbit IgG | Abclonal  ZENBIO  Affinity  Affinity  Affinity  Affinity  Affinity  Affinity  Affinity  Affinity  Affinity  Affinity  Affinity  Zs-BIO  Zs-BIO  Zs-BIO | 55000001847  KK0511  88s1101  21j3698  52e1437  25n2116  87a7182  44q6915  70g0181  19AW0505  34d5362  20t9742  54f8512  51u8853  142637  139931 | 70kDa  60kDa  44kDa  58kDa  44kDa  34kDa  29kDa  21kDa  26kDa  42kDa  56kDa  56kDa  84kDa  84kDa | goat anti-rabbit  goat anti-rabbit  goat anti-rabbit  goat anti-rabbit  goat anti-rabbit  goat anti-rabbit  goat anti-rabbit  goat anti-rabbit  goat anti-rabbit  goat anti-rabbit  goat anti-rabbit  goat anti-rabbit  goat anti-mouse  goat anti-mouse | 1:1000  1:1000  1:1000  1:1000  1:1000  1:500  1:500  1:500  1:1000  1:1000  1:1000  1:500  1:500  1:500 | 10%  10%  10%  10%  10%  10%  10%  10%  10%  10%  10%  10%  10% |

Table S3 Quality control of RNA sequencing

| Sample name | Seq type | Orientation | Raw reads(M) | Raw bases(G) | Q20 ratio(%) |
| --- | --- | --- | --- | --- | --- |
| OE+FTO-1  OE+FTO-2  OE+FTO-3  OE+NC-1  OE+NC-2  OE+NC-3 | mRNA  mRNA  mRNA  mRNA  mRNA  mRNA | Forward/Reverse  Forward/Reverse  Forward/Reverse  Forward/Reverse  Forward/Reverse  Forward/Reverse | 94.45  91.30  87.12  93.59  96.29  94.44 | 14.17  13.7  13.07  14.04  14.44  14.17 | 98.18  97.52  97.98  97.94  97.96  98.15 |

**Table S4 All significantly enriched signaling pathways in KEGG analysis**

| Pathway ID | Pathway description | *P* value | Gene count |
| --- | --- | --- | --- |
| hsa04510  hsa05222  hsa05146  hsa04935  hsa04068  hsa05165  hsa04934  hsa05160  hsa05203  hsa05211  hsa04520  hsa04512  hsa05215  hsa04933  hsa04668  hsa04110  hsa04380  hsa04151  hsa04910  hsa05161  hsa04630  hsa05164  hsa05205 | Focal adhesion  Small cell lung cancer  Amoebiasis  Growth hormone synthesis, secretion and action  FoxO signaling pathway  Human papillomavirus infection  Cushing syndrome  Hepatitis C  Viral carcinogenesis  Renal cell carcinoma  Adherens junction  ECM-receptor interaction  Prostate cancer  AGE-RAGE signaling pathway in diabetic complications  TNF signaling pathway  Cell cycle  Osteoclast differentiation  PI3K-AKT signaling pathway  Insulin signaling pathway  Hepatitis B  JAK-STAT signaling pathway  Influenza A  Proteoglycans in cancer | 0.0003  0.0005  0.0006  0.0010  0.0013  0.0019  0.0021  0.0021  0.0045  0.0059  0.0063  0.0095  0.0114  0.0121  0.0156  0.0191  0.0194  0.0202  0.0220  0.0301  0.0314  0.0332  0.0462 | 4  3  3  3  3  4  3  3  3  2  2  2  2  2  2  2  2  3  2  2  2  2  2 |
